# Studies on the Curing Efficiency and Mechanical Properties of Bis-GMA and TEGDMA Nanocomposites Containing Silver Nanoparticles

**DOI:** 10.3390/ijms19123937

**Published:** 2018-12-07

**Authors:** Izabela Barszczewska-Rybarek, Grzegorz Chladek

**Affiliations:** 1 Department of Physical Chemistry and Technology of Polymers, Silesian University of Technology, 44-100 Gliwice, Poland; 2 Institute of Engineering Materials and Biomaterials, Silesian University of Technology, 44-100 Gliwice, Poland; Grzegorz.Chladek@polsl.pl

**Keywords:** silver nanocomposite, dimethacrylate, molecular structure, flexural properties, water sorption

## Abstract

Bioactive dimethacrylate composites filled with silver nanoparticles (AgNP) might be used in medical applications, such as dental restorations and bone cements. The composition of bisphenol A glycerolate dimethacrylate (Bis-GMA) and triethylene glycol dimethacrylate (TEGDMA) mixed in a 60/40 wt% ratio was filled from 25 to 5000 ppm of AgNP. An exponential increase in resin viscosity was observed with an increase in AgNP concentration. Curing was performed by way of photopolymerization, room temperature polymerization, and thermal polymerization. The results showed that the polymerization mode determines the degree of conversion (*DC*), which governs the ultimate mechanical properties of nanocomposites. Thermal polymerization resulted in a higher *DC* than photo- and room temperature polymerizations. The *DC* always decreased as AgNP content increased. Flexural strength, flexural modulus, hardness, and impact strength initially increased, as AgNP concentration increased, and then decreased at higher AgNP loadings. This turning point usually occurred when the *DC* dropped below 65% and moved toward higher AgNP concentrations, according to the following order of polymerization methods: photopolymerization < room temperature polymerization < thermal polymerization. Water sorption (WS) was also determined. Nanocomposites revealed an average decrease of 16% in *WS* with respect to the neat polymer. AgNP concentration did not significantly affect WS.

## 1. Introduction

**Scheme 1:**
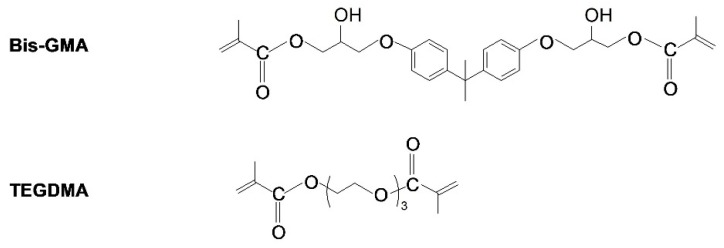
The Bis-GMA and TEGDMA chemical structure.

Recently, polymeric biomaterials with microbiological activity have increasingly gained attention in the potential treatment of various types of inflammation or infection [1]. In dentistry and orthopedy, biomaterials based on dimethacrylates have a superior status, serving as restorative dental materials [2] and bone cements [3,4,5]. The most commonly used monomers of this type are: 2,2′-bis-[4-(2-hydroxy-3-methacryloyloxy propoxy)phenyl]propane (Bis-GMA) and triethylene glycol dimethacrylate (TEGDMA)—a diluting monomer (Scheme 1) [2,3,4,5]. Their polymerization results in a highly crosslinked composite matrix [6,7]. One of the negative consequences of this process is polymerization shrinkage, which causes interfacial gap formation. This phenomenon is responsible for the microleakage and accumulation of bacteria beneath and around the reconstruction, causing tissue inflammation and recurrent caries [8,9,10].

It is widely recognized that the interaction of a restorative material with microorganisms is important for its longevity and effectiveness [9]. It might be improved by the incorporation of antimicrobial substances into tooth reconstruction or bone cement [10,11,12]. The group of *Streptococcus mutans* has been described as the most important bacteria related to the formation of dental caries [10,13,14]. Complications of bone replacements are, in turn, primarily caused by staphylococci, in particular, *Staphylococcus aureus* [15]. Faced with such problems, the need to further develop dental restorative materials, adhesives, and bone cements containing antimicrobial agents has arisen.

A variety of nanoparticles are currently under investigation in order to improve the antibacterial properties of biomaterials. Polymeric nanocomposites filled with nanoparticles, such as calcium phosphate [16,17], zinc oxide [13,18,19], titanium dioxide [20], gold [21,22], and silver (AgNP) [13,16,23,24,25,26,27,28] have shown promising capabilities in reducing bacterial proliferation, thereby preventing the degradation of tissue and restoration.

AgNP advantages, such as a wide spectrum and long-term antibacterial activity, low toxicity, and good biocompatibility with human cells have led to its application in medical products, such as wound dressings, catheters, and prostheses [28]. AgNP has also been applied in several areas of dentistry [24,25,26,27,28,29] and orthopedy [15,23,26]. Literature provides much evidence for the antibacterial action of AgNP-containing dental materials against oral streptococci [13,26,27,28]. On the other hand, bone cements filled with AgNP exhibited antimicrobial activity against staphylococci, such as *Staphylococcus aureus*, *Staphylococcus epidermidis*, and *Acinetobacter baumannii* [23,26]. The decrease in the development of recurrent caries and formation of bacterial biofilm on tooth restoration [16,27,30] and bone cementation [23] was achieved by the introduction of AgNP at the concentration of 500 to 5000 ppm.

Despite evident benefits of nanocomposites with microbiological activity, their potential is limited by the nanofiller agglomeration process at a high nanofiller volume fraction. If the nanofiller content exceeds a critical value, the polymerization extent decreases, which leads to a decrease in polymer network homogeneity [31]. The number of pendant groups, pendant chains, or even free monomer molecules within the poly(dimethacrylate) matrix increases. Consequently, macroscopic properties of the composite are negatively influenced [32]. Additionally, the elution of unreacted monomer from a reconstruction may negatively influence the biological compatibility of the applied biomaterial. The literature has revealed that the greater the content of nanoparticles in the composite, the greater the amount of monomers in the eluate [29].

In view of the above, knowledge regarding structure-property relationships within nanocomposites represents a crucial aspect. The literature, dealing with dimethacrylate systems filled with AgNP, is still lacking a thorough study on this issue.

The purpose of this study was to characterize polymer nanocomposites obtained by photochemical, room temperature, and thermal polymerizations of Bis-GMA/TEGDMA 60/40 wt% composition enriched with silver nanoparticles (AgNP). The AgNP content was increased from 25 to 5000 ppm. The work focused on differences induced by the polymerization initiation methods and AgNP content on the degree of conversion, mechanical properties, and water sorption. Mechanical examination of nanocomposites included the determination of the modulus of elasticity, flexural strength, hardness, and impact resistance.

## 2. Results

In the present study, the mixture of Bis-GMA and TEGDMA at 60/40 wt% ratio was loaded with AgNP in the quantity of 25 to 5000 ppm. As shown in Table 1, eight Bis-GMA/TEGDMA/AgNP resin compositions were produced using the solvent casting method. n-Hexane was used as a solvent.

**Figure 1:**
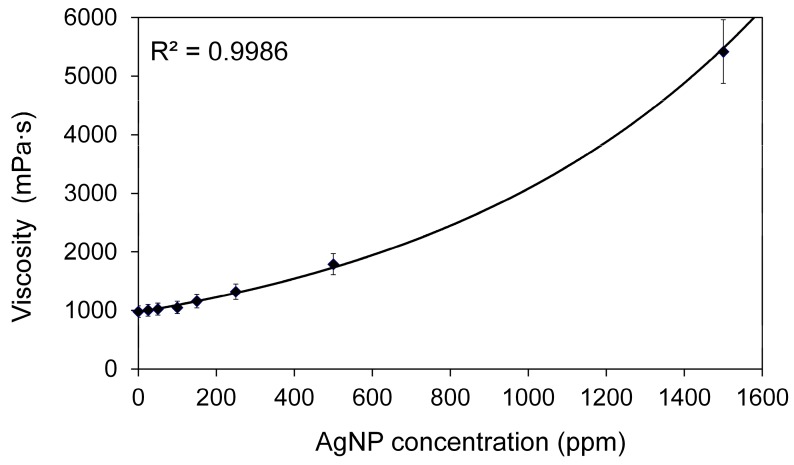
The relationship between viscosity and AgNP concentration in Bis-GMA/TEGDMA/AgNP compositions. All results were statistically significant (*p* < 0.05).

Before polymerization, Bis-GMA/TEGDMA/AgNP compositions, as well as the neat Bis-GMA/TEGDMA mixture, were tested for viscosity. An exponential increase in viscosity with AgNP concentration was observed (Figure 1). 

As shown in Figure 1, by increasing the AgNP concentration from 0 to 1500 ppm, the viscosity increased from 980 to 5420 mPa·s. Above 1500 ppm of AgNP, the viscosity increased dramatically, which necessitated the use of another torque in further studies. Consequently, 98 mPa·s was determined for the sample filled with 5000 ppm of AgNP. This value could not be compared with the previous results, found using the torque designed for lower viscosities. Thus, this value was not taken into account when the correlation between viscosity and AgNP concentration was constructed.

The monomer mixtures were then activated for polymerization in three ways:

1. Photopolymerization—by the addition of CQ (camphorquinone) 0.4 wt.% and DMAEMA (N,N-dimethylaminoethyl methacrylate) 1 wt.%;
2. Room temperature polymerization—by the addition of BPO (benzoyl peroxide) 0.5 wt% and DMPT (N,N-dimethyl-p-toluidine) 0.05 wt%;
3. Thermal polymerization—by the addition of BPO 1 wt%.

Macroscopic observations led to the first conclusion that the polymerization initiation mechanism restricts the AgNP capacity in the nanocomposite. 

**Figure 2:**
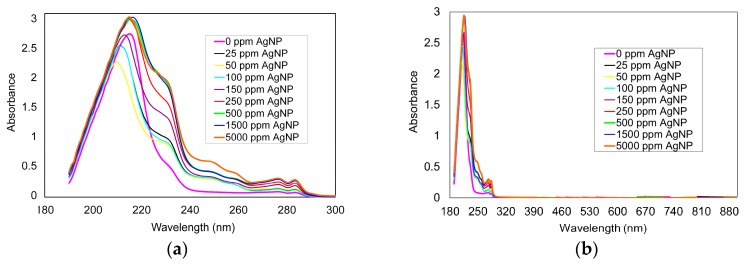
UV/VIS spectra of the pristine Bis-GMA/TEGDMA and its AgNP loaded modifications in the range of: (**a**) 190–300 nm; (**b**) 190–900 nm.

Photopolymerization was limited by 250 ppm of AgNP. A higher AgNP content resulted in a poor sample quality, which was mechanically weak and brittle. The absorption of UV-VIS radiation was suspected as the key factor responsible for this limitation. To check this hypothesis, UV-VIS analysis was performed on the neat Bis-GMA/TEGDMA composition, as well as its AgNP loaded modifications. As shown in Figure 2, all samples were likely to absorb UV-VIS radiation within the range of 190 to 300 nm, with the maximum at around 215 nm. However, the absorption intensity of nanocomposites was higher than the absorption intensity of the neat network. The higher the AgNP quantity, the higher the absorption intensity. This region corresponds to the CQ absorption in the UV region [33]. Additionally, CQ absorbs electromagnetic radiation in the VIS region from 400 to 550 nm, with the maximum absorption intensity at 467 nm [33]. This part of the spectra remained free from absorption bands of Bis-GMA/TEGDMA/AgNP (Figure 2b). 

In the case of room temperature polymerization, 1500 ppm of AgNP was the maximum concentration that could be employed to maintain a good visual quality of the sample. The introduction of 5000 ppm of AgNP caused an enormous increase in the viscosity of the resinous system. This prevented the escape of air bubbles before the polymer was hardened. Consequently, a significant weakening of the nanocomposite was observed.

Thermal initiation was successfully adopted to produce nanocomposites of a high quality and durability over the whole AgNP concentration range.

Following the initial sample quality assessment, the AgNP concentration range was narrowed individually for each group of nanocomposites according to the polymerization technique. Thus, the following maximum AgNP concentrations were set up in a sample preparation for further studies: 250 ppm—photopolymerization, 1500 ppm—room temperature polymerization, and 5000 ppm—thermal polymerization. The list of tested samples and their names are presented in Table 2.

In Table 3, the results for the degree of conversion (*DC*) and polymerization shrinkage (*S*) are summarized.

The *DC* in the neat networks produced by photo- and room temperature polymerizations was almost the same and equaled 70%. The *DC* in the thermally polymerized neat network was higher and equaled 78%. However, a statistically significant difference was only found for the RT0 and T0 couple. The average polymerization shrinkage of the neat networks was 8.4% and there was no statistically significant difference between the Ph0, RT0, and T0 samples. 

The analysis of results for nanocomposites showed that the introduction of AgNP into the Bis-GMA/TEGDMA matrix resulted in a decrease of the *DC* and *S* (polymerization shrinkage). The higher the AgNP concentration, the lower the *DC* and *S.* It is worth noting that the *DC* in T5000 did not achieve a satisfactory degree of conversion, which was 43.6%. Statistically significant decreases in the *DC*, with respect to the neat networks, were found, beginning from the following AgNP concentrations: 150 ppm—for photopolymerization, 500 ppm—for room temperature polymerization, and 5000 ppm—for thermal polymerization. An analogous analysis of the results for *S* allowed for the specification of the following AgNP concentrations, which resulted in statistically significant decreases in *S*, with respect to the neat networks, respectively: 250 ppm, 500 ppm, and 1500 ppm. 

A detailed analysis of the *DC* and *S* led to the conclusion that both parameters increased by the initiation mechanism according to the following order: photopolymerization < room temperature polymerization < thermal polymerization. The *DC* in nanocomposites obtained by thermal polymerization was usually statistically significantly higher than the *DC* in nanocomposites obtained by photopolymerization and room temperature polymerization.

In Table 4, the results for flexural properties are summarized. It may be seen that the flexural strength (*σ*) and flexural modulus (*E*) depend on the AgNP concentration.

The flexural strength of the neat networks derived from room temperature polymerization (79.8 MPa) and photopolymerization (88.6 MPa) was lower than that from thermal polymerization (100.3 MPa). The results for only the RT0 and T0 couple were statistically significant. The elasticity of the pristine networks was similar (*p* ≥ 0.05) and it was characterized by the average *E* value of 3767.5 MPa. By filling Bis-GMA/TEGDMA with AgNP, increases in both elastic properties were observed. The flexural strength of nanocomposites produced by photopolymerization increased by 11%, whereas the modulus increased by 6%. *σ* of samples produced by room temperature polymerization increased by 23%, whereas *E* increased by 11%. Thermally activated polymerizations resulted in the greatest increases in *σ* and *E*, which correspond, respectively, to 29 and 19%. The detailed analysis of results for *σ* and *E* within particular series, led to the observation that their values increased with an increasing AgNP content at the beginning (low AgNP concentrations), but then decreased with an increasing AgNP content at high AgNP concentrations. The maximum values of *σ* were noted for the following nanocomposites: Ph50, RT100, and T250, whereas the maximum values for *E* were found for: Ph50, RT100, and T1500. Statistically significant drops in *σ* with respect to the pristine networks were recorded for the first time for Ph150 and T5000. *σ* of none of the chemically cured nanocomposites revealed statistically significant decreases with respect to RT0. The first statistically significant drop in *E*, with respect to Ph0, was recorded for Ph250, whereas with respect to RT0, it was recorded for RT1500. None of the thermally cured samples showed statistically significant decreases in *E* with respect T0. This result may be due to the fact that in thermally activated samples, the *DC* was relatively high (in most cases it was higher than 70%) compared to Ph or RT samples.

When comparing the elastic properties of nanocomposites by the polymerization method, the following order of increasing *σ* and *E* might be constructed: photopolymerization < room temperature polymerization < thermal polymerization. Statistical analysis showed that thermally polymerized materials had a significantly higher bending strength than products of photo- and room temperature polymerizations. The same tendency could be found by comparing results for modulus; however, they did not usually show statistical significance. 

The decreases in *σ* and *E*, observed with increasing AgNP concentration in nanocomposites, coincide with the decreasing *DC*. The comprehensive analysis of changes in the *DC*, *σ*, and *E* led to the conclusion that AgNP in the Bis-GMA/TEGDMA/AgNP nanocomposites can play a reinforcing role, by increasing *σ* and *E*, when the *DC* is at a minimum of about 65%.

In Table 5, the results for the hardness and impact strength of studied materials are summarized.

The hardness (*H*) of pristine networks did not differ significantly and the average value equaled 115.7 N/mm^2^. In the composite series, the values of *H* increased to a certain AgNP limiting content and afterwards, they decreased. Thus, the following nanocomposites were characterized by the highest hardness in the series: Ph50, RT150, and T5000, which correspond to the following percentage increases, respectively: 7%, 18%, and 30%. Statistically significant decreases in *H*, with respect to pristine polymers, were found for Ph250 and RT1500, i.e., for nanocomposites with the highest AgNP loadings in the photo- and room temperature polymerized series. The hardness of the thermally polymerized samples increased over the whole concentration range. Moreover, the T5000 nanocomposite was characterized by the highest hardness noted in this study.

When comparing the hardness of nanocomposites according to the initiation mechanism, the following increasing order can be constructed: photopolymerization < room temperature polymerization < thermal polymerization. However, these results were usually not statistically significant. 

When comparing the impact strength of pristine polymers by the polymerization technique, the values might be ordered accordingly: room temperature polymerization (4.07 kJ/m^2^) < photopolymerization (4.57 kJ/m^2^) < thermal polymerization (5.20 kJ/m^2^). Statistically significant results were produced for the RT0 and T0 couple. The same order of polymerization techniques might be constructed if *a_n_* of nanocomposites was analyzed. The results for nanocomposites obtained by thermal polymerization were statistically significantly higher compared to results for their counterpart samples produced in photo- or chemically initiated processes.

By comparing results in the nanocomposite series, it can be seen that the impact strength increased as the AgNP content increased up to a certain limit and then decreased, with a further increase of AgNP concentration. The impact resistance of photopolymerized samples reached the maximum in Ph50, which corresponded to 5.5% income with respect to Ph0. The impact resistance (*a_n_*) of samples polymerized on the chemical route increased by 4.9%, which was recorded for RT100. Thermal polymerization resulted in a 6.5% increase in *a_n_*, which was achieved for T100. All these increases were statistically insignificant with respect to the pristine polymers. Statistically significant drops in *a_n_* of nanocomposites, with respect to the pristine polymers, were noted for Ph250, RT500, and T500. 

**Figure 3:**
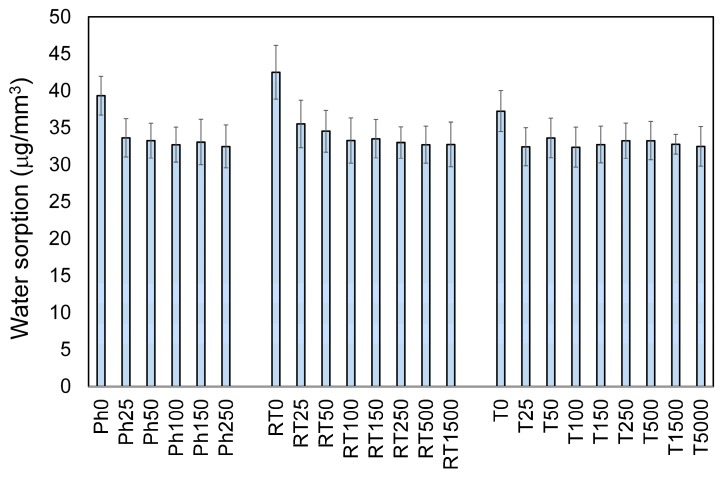
Water sorption of Bis-GMA/TEGDMA/AgNP nanocomposites. The statistically significant results were obtained between pristine polymers and nanocomposites in each series (*p* < 0.05).

In Figure 3, the results for water sorption (WS) are shown. It may be seen that AgNP incorporation caused the decrease in the values by around 16.5%. The average WS of nanocomposites was 33.2 μg/mm^3^, whereas the WS of pristine polymers equaled 37.7 μg/mm^3^. The polymerization technique did not significantly influence WS (*p* ≥ 0.05). 

## 3. Discussion

The present paper describes research regarding the effect of AgNP concentration in the Bis-GMA/TEGDMA 60/40 wt% on the viscosity of its liquid form, as well as structural, physical, and mechanical properties of hardened materials. 

The results showed that AgNP concentration influences Bis-GMA/TEGDMA/AgNP resin viscosity, which increases as the AgNP content increases. An exponential increase in viscosity resulted in the enormously limited flowability of the mixture filled with 5000 ppm of AgNP. This means that the concentration of AgNP in the Bis-GMA/TEGDMA/AgNP mixture should not be higher than 1500 ppm. A higher AgNP concentration may considerably restrict resin flowability, which gives rise to air bubble trapping and can exclude the possibility of introducing other fillers, typically used in dental composites and bone cements to improve their mechanical characteristics. 

The structure-property relationships were tested for Bis-GMA/TEGDMA/AgNP nanocomposites obtained by radical polymerization of the dimethacrylate matrix, which was initiated according to three mechanisms:

1. Photopolymerization—commonly used for direct curing of dental restorations [2];
2. Room temperature chemical polymerization—commonly used in bone cementation [3];
3. Thermal polymerization—commonly used in industrial processes for the manufacturing of thick parts [34].

The neat networks were characterized by the *DC* from 70 to 78%, which is in agreement with the literature data. The polymerization of dimethacrylates is never complete. A considerable fraction of the double bonds remains unreacted because of immobilization, caused by a set of certain complex features, such as autoacceleration, autodeceleration, steric isolation, and vitrification [6,7,35,36,37,38]. This has been demonstrated in many studies on the photopolymerization [35,36], room temperature polymerization [37], and thermal polymerization [38] of Bis-GMA/TEGDMA systems. The *DC* of 78% was the highest measured in this study and corresponded to the thermally polymerized sample. It can be explained by the effects of temperature on the polymerization kinetics. It is well-known that the higher polymerization temperature, the higher the final conversion in the dimethacrylate network. Heating provides the energy to improve the segmental mobility of the polymer chain and makes more residual unsaturation sites accessible for polymerization [35,36]. Additionally, the thermodynamic miscibility of Bis-GMA and TEGDMA monomers is greater at elevated temperatures. They can undergo phase separation at room temperature, which can result in the formation of more heterogeneous networks [36]. In the early stage of the thermal polymerization process, the temperature was kept at 40 °C for 1 h. At this temperature, the BPO decomposition rate was slow enough that the system nearly did not polymerize [39] and the viscosity decreased sufficiently enough to allow air bubbles to escape. Due to the preheating of liquid samples, high macroscopic homogeneity of the cured materials was maintained, despite the increase in viscosity. Gradually raising the temperature to 100 °C, in combination with a 24 h total polymerization time, resulted in materials of the highest structural homogeneity and best mechanical performance. The temperature of 100 °C was higher than the glass temperature of the Bis-GMA/TEGDMA polymer network [36]. It was chosen to intensify segmental mobility during post-curing.

The results for the *DC* in nanocomposites showed that it depends on the AgNP concentration, as well as the polymerization initiation mechanism. 

The decrease in *DC* with the increase of AgNP concentration is likely caused by the increasing distance between double bonds. The free volume in the nanocomposite is occupied by the AgNP agglomerates. The higher the AgNP concentration, the higher the tendency of forming larger agglomerates [31]. As the dimensions of agglomerates and their number increase, the reactive groups drift further apart and thus become less available and less reactive. 

The following general order of increasing *DC* according to polymerization methods: photopolymerization < room temperature polymerization < thermal polymerization, results from a variety of factors. The poorest curing efficiency of photopolymerization might be explained by the presence of partially overlapping absorption of Bis-GMA/TEGDMA/AgNP and CQ in the UV region. Even though the UV/VIS spectrum was free from any absorption bands from Bis-GMA/TEGDMA/AgNP in the VIS region, where CQ absorbs in the 400–550 nm range, the curing efficiency in nanocomposites produced by photopolymerization was insufficient when AgNP content exceeded 250 ppm. The effectiveness of room temperature polymerization was most likely restricted by the excessive high viscosity of the resin system, which causes the trapping of air bubbles. The highest *DC* of nanocomposites manufactured by thermal polymerization can be explained by the same kinetic factors as mentioned above. However, it has to be noted that the T5000 nanocomposite was characterized by the *DC* of 44%. Since the *DC* lower than 50% gives the information of free monomer occurrence in the system, 1500 ppm might be proposed as a maximum AgNP concentration in possible potential applications of studied nanocomposites [40].

Since *S* is the consequence of the *DC*, as predicted, the same trend in *S* values was observed with increasing AgNP content and polymerization technique. 

**Figure 4:**
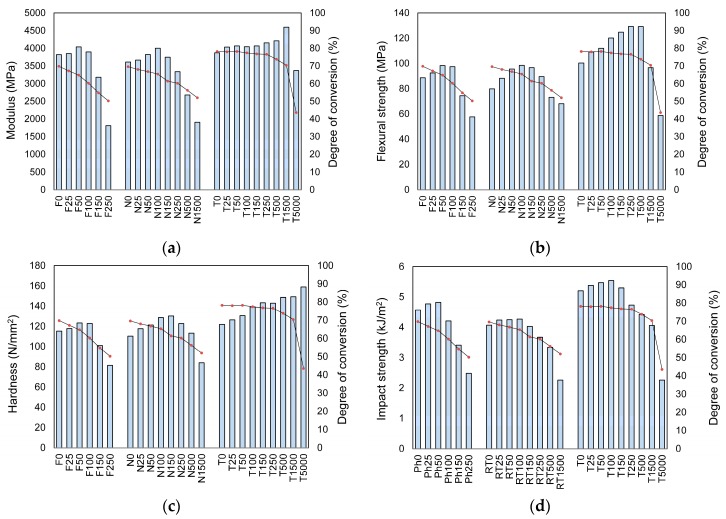
The comparison of mechanical properties with the degree of conversion: (**a**) modulus; (**b**) flexural strength; (**c**) hardness; (**d**) impact resistance. Bar charts correspond to mechanical properties, whereas line charts correspond to the *DC*.

The results also showed that mechanical properties of Bis-GMA/TEGDMA/AgNP nanocomposites mainly depend on the *DC* in the dimethacrylate polymer matrix, which is governed by the initiation mechanism (Figure 4). 

Thermal polymerization resulted in a higher flexural strength, modulus, and hardness than photopolymerization and room temperature polymerization. Impact strength increased accordingly: room temperature polymerization < photopolymerization < thermal polymerization. The top position of thermally polymerized nanocomposites in these arrangements might be attributed to their maximum *DC* and the best sample quality. The lowest *a_n_* of chemically polymerized nanocomposites might be attributed to lower *DC* and a larger number of air bubbles trapped in the sample. Since the room temperature polymerization begins just after mixing the components of the initiation system, the resin viscosity increases radically in a short time and perfect trapped air removal is unattainable. For this reason, bone cements typically display an overall porosity ranging from 2 to 18% [41,42]. This means that the latter property may be considerably deteriorated by air trapped bubbles, which are specific for room temperature polymerization.

As can be seen from Figure 4, mechanical properties usually increased at the beginning, at lower AgNP concentrations, and then decreased, when AgNP concentrations exceeded a certain level. The decreases in *σ*, *E*, *H*, and *a_n_*, which were observed with increasing AgNP concentration in nanocomposites, coincide with the decreasing *DC*. This result coincides with previous findings, which show that the modulus, mechanical strength, and hardness decrease as the degree of conversion decreases [6,7]. The detailed analysis of changes in the *DC*, *σ*, and *E* leads to the conclusion that AgNP in the Bis-GMA/TEGDMA/AgNP nanocomposites can play a reinforcing role on the condition that the *DC* is at a minimum of 65%. In the series of nanocomposites obtained by thermal polymerization, hardness increased throughout the series, regardless of the degree of conversion. Therefore, hardness might be considered as the least sensitive to the *DC* property.

Taking the above considerations into account, the following AgNP concentrations might be recommended as the upper limits for manufacturing Bis-GMA/TEGDMA/AgNP nanocomposites with a satisfactory physico-mechanical performance: 250 ppm—for photopolymerization and 1500 ppm—for room temperature and thermal polymerizations. 

The water sorption of the nanocomposites studied decreases with the mere addition of AgNP by an average of 16% and an increase in its concentration does not significantly influence the WS behavior. This result might be attributed to the hydrophobic character of AgNP [43].

## 4. Materials and Methods 

### 4.1. Materials

The dimethacrylate resins: Bis-GMA and TEGDMA, components of the initiation systems: CQ (camphorquinone), DMAEMA (*N*,*N*-dimethylaminoethyl methacrylate), and DMPT (*N*,*N*-dimethyl-*p*-toluidine) were purchased from Sigma-Aldrich (St. Louis, MO, USA) and used as received. The BPO initiator—(benzoyl peroxide) came from POCh (Gliwice, Poland) and it was purified by dissolving in chloroform (POCh, Gliwice, Poland) and precipitated by adding methanol (POCh, Gliwice, Poland). n-Hexane was purchased from POCh (Gliwice, Poland) and was used as received. 

AgNP colloid in hexane of the 1000 ppm concentration was purchased from AMEPOX Co. Ltd. (Lodz, Poland). The AgNP/hexane colloid was used as received or diluted with n-hexane to the concentrations of 500 and 100 ppm (Table 1). The mean particle size was 22.8 nm, as measured in a previous study [44]. 

### 4.2. Preparation of AgNP-Loaded Resin Compositions 

The monomer blend consisted of Bis-GMA and TEGDMA in a 60/40 wt% ratio. It was loaded with AgNP. Eight concentrations of AgNP: 25, 50, 100, 150, 250, 500, 1500, and 5000 ppm, were prepared by solvent casting, with the use of n-hexane as a solvent.

First, 40 g of TEGDMA was dissolved in 100 mL of n-hexane. Next, the precisely measured amount of AgNP/hexane colloid was added to the TEGDMA solution in n-hexane (Table 1). Mixing was performed in an Erlenmayer flask on a magnetic stirrer for 10 min. Hexane was evaporated from the TEGDMA/AgNP/hexane dispersion in a rotary evaporator (IKA RV-10, Staufen, Germany) for 20 min, under the pressure of 50 mbar and then for 20 min, under the pressure of 5 mbar. The effectiveness of the evaporation procedure was confirmed in the ^1^H NMR experiments (UNITY INOVA, Varian, 300 MHz, Palo Alto, CA, USA), which did not show the presence of peaks corresponding to n-hexane on 1H NMR spectra. TEGDMA loaded with AgNP was then blended with 60 g of Bis-GMA. Mixing was performed in a glass vessel with a mechanical stirrer at 40 °C for 20 min. 

### 4.3. Curing Procedure 

Polymerizations were carried out in 12 cm diameter Petri dishes and 10 mm diameter Teflon O-rings placed on a glass surface. The polymer amounted to the obtained thicknesses of 2 and 1 mm, respectively. 

#### 4.3.1. Photopolymerization 

Bis-GMA/TEGDMA/AgNP liquid compositions, loaded with AgNP in the concentration specified in Table 1 were mixed with 0.4 wt.% of CQ (initiator) and 1 wt.% of DMAEMA (reducer) and poured into molds. Before irradiation, the sample surface was covered with a PET (poly(ethylene terephtalate)) film in order to avoid the oxygen inhibition effect. The mixtures were irradiated with a mercury UV/VIS lamp (FAMED-1, Zywiec, Poland, power 375 W) set at a distance of 10 cm for 15 min.

#### 4.3.2. Room Temperature Polymerization (Chemical Polymerization)

Bis-GMA/TEGDMA/AgNP liquid compositions, loaded with AgNP in the concentration specified in Table 1, were divided into two equimass portions. One of them was mixed with 0.5 wt% of BPO (radical polymerization initiator) and the other with 0.05 wt% of DMPT (redox activator for BPO). To perform curing, both components were thoroughly mixed for 60 s. After stirring, the mixtures were poured into molds, covered with PET film, and left for 24 h. 

#### 4.3.3. Thermal Polymerization

Bis-GMA/TEGDMA/AgNP liquid compositions, loaded with AgNP in the concentration specified in Table 1, were mixed with 1 wt% of BPO, poured into molds, and polymerized in a drying oven under nitrogen—reducing oxygen inhibition, by raising the temperature gradually from 40 to 100 °C over 24 h [40]. First, the polymerizing mixture was reheated at 40 °C for 1 h; next, the temperature was increased to 70 °C and maintained at that temperature overnight; and finally, the temperature was increased to 100 °C and maintained for 2 h. 

### 4.4. UV/VIS Spectroscopy 

The UV/VIS spectra of pristine Bis-GMA/TEGDMA monomer composition, as well as its modifications with AgNP, were recorded over a wavelength ranging from 190 to 500 nm, utilizing a UV/VIS spectrometer (UV 2600, Shimadzu Co., Kyoto, Japan) with a 10-cm path-length quartz cuvette. 

### 4.5. Viscosity

The viscosity (η, mPa·s) of liquid Bis-GMA/TEGDMA and its compositions with AgNP was measured by means of a rotating spindle viscometer (Brookfield Fungilab Viscometer, Visco Star Plus L, Barcelona, Spain) at 25 °C, according to PN-ISO 2555 (Plastics—Resins in the liquid state or as emulsions or dispersions—Determination of apparent viscosity by the Brookfield method). Viscosity was measured using the appropriate spindle, which allowed for recording viscosity values between 10% and 90% torque. Compositions containing 0 to 1500 ppm AgNP were tested utilizing the same spindle of the designation “1”. The composition containing 5000 ppm was characterized by a significantly higher viscosity, which required the use of a spindle with the designation of “3”. 

### 4.6. Polymerization Shrinkage

The density of resinous compositions (*d_m_*) was measured utilizing a liquid pyknometer at 25 °C according to ISO 1675 (Plastics—Liquid resins—Determination of density by the pyknometer method). Their cured forms were tested for density (*d_p_*) according to Archimedes’ principle, on the Mettler Toledo XP Balance with a 0.01 mg accuracy (Greifensee, Switzerland) with the density determination kit at 25 °C. Water was used as the immersing liquid. The volumetric shrinkage (*S*) was determined by the following equation:

(1) $$S\left(\%\right)=\frac{d_{p}-d_{m}}{d_{p}}\times 100$$

### 4.7. Degree of Conversion

The degree of conversion (*DC*) in the polymer matrix of studied nanocomposites was determined by using an FTIR spectrophotometer (Bio-Rad Laboratories, FTS 175C, Hercules, CA, USA). The spectra of the monomers and their polymers were recorded with 128 scans at a resolution of 1 cm^−1^. The monomer samples were tested as very thin films on KBr pellets. The cured samples were pulverized into fine powder with a particle diameter of less than 24 μm and analyzed as KBr pellets. The *DC* was calculated from the decrease of the absorption band at 1637cm^−1^, referring to the C=C stretching vibration (*A_C=C_*), in relation to the peak at 1609 cm^−1^ assigned to aromatic stretching vibrations (*A_Ar_*) [45]:

(2) $$DC\left(\%\right)=\left(1-\frac{{\left(A_{C=C}/A_{Ar}\right)}_{polymer}}{{\left(A_{C=C}/A_{Ar}\right)}_{monomer}}\right)\times 100$$

### 4.8. Mechanical Properties

#### 4.8.1. Flexural Properties 

The flexural modulus (*E*) and flexural strength (*σ*) were determined in accordance with ISO 178 (Plastics—Determination of flexural properties) in three-point bending tests, using a universal testing machine (Zwick Z020, Ulm, Germany). The rectangular samples (length × width × thickness: 40 mm × 10 mm × 2 mm) were tested. *E* and *σ* were calculated according to the following equations:

(3) $$E\;\left(\mathrm{MPa}\right)=\frac{P_{1}l^{3}}{4bd^{3}\delta }$$

(4) $$\sigma \;\left(\mathrm{MPa}\right)=\frac{3Pl}{2bd^{2}}$$

where, *P* is the maximum load, *P*_1_ is the load at a selected point of the elastic region of the stress–strain plot, *l* is the distance between supports, *b* is the specimen width, *d* is the specimen thickness, and *δ* is the deflection of the specimen at *P*_1_. 

#### 4.8.2. Hardness 

The ball indentation hardness (*H*) was determined according to ISO 2039-1 (Plastics—Determination of hardness—Part 1: Ball indentation method) on the VEB Werkstoffprüfmaschinen apparatus (Leipzig, Germany). The 4 mm-thick disc-like samples were prepared by stacking two 2 mm layers. H was calculated according to the following equation:

(5) $$H\;\left(\mathrm{MPa}\right)=\frac{F_{m}\left(\frac{0.21}{h-h_{r}+0.21}\right)}{\pi dh_{r}}$$

where, *h_r_* is the reduced depth of impression (*h_r_* = 0.25 mm), *d* is the diameter of the ball indenter (*d* = 5 mm), *F_m_* is the test load on the indenter, and *h* is the depth of impression.

#### 4.8.3. Impact Strength 

The impact strength (*a_n_*) was determined in accordance with DIN 53435 (Testing of plastics. Bending test and impact test on Dynstat test pieces) using VEB Werkstoffprüfmaschinen Dynstat apparatus on rectangular unnotched specimens (length × width × thickness: 15 mm × 10 mm × 2 mm). The following formula was applied to calculate *a_n_*:

(6) $$a_{n}\;\left(\frac{\mathrm{kJ}}{m^{2}}\right)=\frac{A_{n}}{bd}$$

where, *A_n_* is the impact energy required to cause a material to fracture, and *b* and *d* are the width and thickness of the specimen, respectively.

### 4.9. Water Sorption

Water sorption was measured according to ISO 4049 (Dentistry—Polymer-based restorative materials). Disc-like specimens (diameter × thickness: 10 mm × 1 mm) of each nanocomposite were dried in a pre-conditioning oven at 37 °C until their weight was constant. This result was recorded as m_0_ (Mettler Toledo XP Balance with 0.01 mg accuracy, Greifensee, Switzerland). The specimens were then immersed in distilled water and maintained at 37 °C for a week. After this time, the samples were removed, blotted dry, and weighed (m_1_). Water sorption (WS) was calculated using the following formula:

(7) $$\mathrm{WS}\left(\frac{\mu g}{{\mathrm{mm}}^{3}}\right)=\frac{m_{1}-m_{0}}{V_{0}}$$

where, m_0_ is the initial sample weight, *V*_0_ – the initial volume of the sample.

### 4.10. Statistical Analysis

The experimental results were analyzed using one-way analysis of variance (ANOVA). The pair-wise comparisons were conducted by means of the Student’s *t*-test with a significance level (*p*) of 0.05. For each physical property, a set of five samples was tested. The results of measurements were expressed as mean values with associated standard deviations. 

## 5. Conclusions 

This study explained the AgNP influence on the degree of conversion and mechanical properties of Bis-GMA/TEGDMA/AgNP nanocomposites. It was shown that the higher the AgNP concentration, the lower the *DC*. Among the polymerization methods used, the thermal polymerization resulted in the highest level of curing. 

Further analysis showed that AgNP can have a strengthening effect on mechanical properties. However, their values increased at the beginning, at lower AgNP concentrations, and then decreased, as AgNP concentrations exceeded a certain level, which was varied and dependent on the property. Hardness showed the least sensitivity to the *DC*, followed by the modulus, bending strength, and impact resistance, in that order. The latter property, besides possessing the highest sensitivity to the *DC*, was most likely deteriorated by air trapped bubbles, which are specific for chemical polymerization.

## Figures and Tables

**Table 1:** The AgNP concentrations in Bis-GMA/TEGDMA/AgNP compositions, AgNP concentrations in AgNP/hexane colloids, and colloid amounts used in nanocomposite manufacturing.

AgNP Concentration in Bis-GMA/TEGDMA/AgNP Compositions (ppm)	AgNP Concentration in hexane Colloid (ppm)	The Amount of AgNP/hexane Colloid (g)
25	100	25.0
50	100	50.0
100	100	100.0
150	100	150.0
250	100	250.0
500	100	500.0
1500	500	300.4
5000	1000	502.5

**Table 2:** The Bis-GMA/TEGDMA/AgNP nanocomposite samples studied and their names: Ph (photopolymerization), RT (room temperature polymerization), T (thermal polymerization).

AgNP Concentration in Nanocomposite (ppm)	Sample Name		
	Photopolymerization	Room Temperature Polymerization	Thermal Polymerization
0	Ph0	RT0	T0
25	Ph25	RT25	T25
50	Ph50	RT50	T50
100	Ph100	RT100	T100
150	Ph150	RT150	T150
250	Ph250	RT250	T250
500	-	RT500	T500
1500	-	RT1500	T1500
5000	-	-	T5000

**Table 3:** The polymerization shrinkage and degree of conversion in studied Bis-GMA/TEGDMA/AgNP nanocomposites.

Sample	Polymerization Shrinkage (%)	Degree of Conversion (%)
Ph0	8.37 ± 0.58 ^1^	69.8 ± 6.0 ^31,32^
Ph25	8.22 ± 0.60 ^2,6^	67.2 ± 7.0 ^33^
Ph50	8.01 ± 0.70 ^3,7^	64.8 ± 4.6 ^34,35,f^
Ph100	7.41 ± 0.62 ^4,6^	60.2 ± 4.2 ^g^
Ph150	6.95 ± 0.96 ^5,7,a,b^	54.8 ± 5.6 ^31,34,h^
Ph250	5.44 ± 0.53 ^1–5,c,d^	50.2 ± 7.5 ^32,33,35,i,j^
RT0	8.39 ± 0.60 ^8,9^	69.6 ± 5.4 ^36,39,k^
RT25	8.13 ± 0.70 ^10^	68.0 ± 4.9 ^37,40^
RT50	7.99 ± 0.62 ^11^	66.8 ± 4.7 ^41,l^
RT100	7.84 ± 0.44 ^12^	65.4 ± 7.2 ^38,42^
RT150	7.77 ± 0.78 ^13,a^	61.4 ± 7.2 ^m^
RT250	7.66 ± 0.73 ^14,c^	60.2 ± 6.3 ^j,n^
RT500	7.41 ± 0.62 ^8,15^	56.2 ± 7.7 ^36–38,o^
RT1500	5.34 ± 0,59 ^9–15,e^	52.0 ± 9.0 ^39–42,p^
T0	8.50 ± 0.50 ^16,23^	78.2 ± 5.5 ^43,k^
T25	8.37 ± 0.43 ^17,24^	78.0 ± 5.7 ^44^
T50	8.28 ± 0.57 ^18,25^	78.2 ± 5.4 ^45,f,l^
T100	8.19 ± 0.48 ^19,26^	77.4 ± 7.0 ^46,g^
T150	8.13 ± 0.69 ^20,27,b^	76.8 ± 7.0 ^47,h,m^
T250	8.11 ± 0.51 ^21,28,d^	76.6 ± 7.2 ^48,i,n^
T500	8.01 ± 0.70 ^22,29^	73.8 ± 4.9 ^49,o^
T1500	7.15 ± 0.74 ^16–22,30,e^	70.4 ± 7.4 ^50,p^
T5000	5.01 ± 0.99 ^23–30^	43.6 ± 7.8 ^43–50^

^1–50^*p* < 0.05—statistically significant results within the nanocomposite series. ^a–p^ *p* < 0.05—statistically significant results between nanocomposites, having the same AgNP content, but obtained in different polymerization modes.

**Table 4:** The flexural properties of studied Bis-GMA/TEGDMA/AgNP nanocomposites.

Sample	Flexural Strength (MPa)	Flexural Modulus (MPa)
Ph0	88.6 ± 9.1 ^1,5^	3819.4 ± 255.8 ^40^
Ph25	92.4 ± 11.7 ^2,6,a^	3848.5 ± 210.5 ^41,45^
Ph50	98.3 ± 11.7 ^3,7,b^	4039.2 ± 361.9 ^42,46^
Ph100	97.4 ± 8.7 ^4,8,c^	3897.2 ± 314.3 ^43,47^
Ph150	74.4 ± 7.7 ^1–4,9,d,f^	3179.6 ± 343.6 ^44,45–47,p^
Ph250	57.6 ± 6.8 ^5–9,e,g^	1808.2 ± 254.9 ^40–44,q,r^
RT0	79.8 ± 10.2 ^10,h^	3610.5 ± 316.0 ^48,54^
RT25	88.2 ± 9.8 ^11,16,i^	3665.6 ± 248.6 ^49,55^
RT50	95.5 ± 9.0 ^12,17,j^	3822.3 ± 292.7 ^50,56,61^
RT100	98.5 ± 10.4 ^13,18,k^	4001.6 ± 442.3 ^51,57,62^
RT150	96.7 ± 10.8 ^10,14,19,a,f,l^	3746.8 ± 290.3 ^52,58^
RT250	89.6 ± 5.9 ^15, 20,c,g,m^	3336.5 ± 307.4 ^53,59,61,62,q,s^
RT500	73.1 ± 7.8 ^11–15,n^	2676.8 ± 227.8 ^47–53,60,t^
RT1500	68.1 ± 7.1 ^16–20,o^	1905.9 ± 289.8 ^54–60,u^
T0	100.3 ± 6.8 ^21,25,h^	3872.5 ± 297.9
T25	109.0 ± 11.0 ^26,27,33,a,i^	4034.0 ± 437.6
T50	112.0 ± 12.5 ^28,34,b,j^	4066.4 ± 415.0
T100	120.16 ± 8.4 ^21,29,35,c,k^	4044.4 ± 272.6 ^61^
T150	124.8 ± 6.3 ^22,30,36,d,l^	4066.5 ± 407.3 ^p^
T250	129.3 ± 15.8 ^23,26,31,37,e,m^	4153.5 ± 303.4 ^62,r,s^
T500	129.2 ± 11.2 ^24,32,38,n^	4210.6 ± 396.6 ^63,t^
T1500	96.6 ± 7.2 ^27–32,39,o^	4596.1 ± 590.6 ^64,u^
T5000	58.7 ± 6.0 ^25,33–39^	3368.9 ± 479.4 ^61–64^

^1–64^*p* < 0.05—statistically significant results within the nanocomposite series. ^a–u^ *p* < 0.05—statistically significant results between nanocomposites, having the same AgNP content, but obtained in different polymerization modes.

**Table 5:** Hardness and impact resistance of studied Bis-GMA/TEGDMA/AgNP nanocomposites.

Sample	Hardness (N/mm^2^)	Impact Resistance (kJ/m^2^)
Ph0	115.1 ± 9.5 ^1^	4.57 ± 0.35 ^26,30^
Ph25	117.9 ± 6.1 ^2^	4.77 ± 0.36 ^27,31^
Ph50	123.3 ± 16.4 ^3,6^	4.82 ± 0.47 ^28,32^
Ph100	122.8 ± 10.6 ^4,7^	4.21 ± 0.45 ^29,33,g^
Ph150	100.9 ± 12.1 ^5,6,7,a,b^	3.41 ± 0.35 ^26–29,34,h^
Ph250	81.4 ± 7.3 ^1–5,c,d^	2.48 ± 0.31 ^30–34,i,j^
RT0	110.2 ± 7.6 ^8,9^	4.07 ± 0.37 ^35,40,k^
RT25	117.7 ± 4.2 ^10^	4.24 ± 0.44 ^36,41,l^
RT50	121.3 ± 6.5 ^11^	4.25 ± 0.42 ^37,42,m^
RT100	128.5 ± 13.1 ^12^	4.27 ± 0.49 ^38,43,n^
RT150	130.2 ± 15.1 ^8,13,16,a^	4.03 ± 0.44 ^39,44,o^
RT250	122.7 ± 14.5 ^14,c^	3.67 ± 0.36 ^45,j,p^
RT500	113.1 ± 11.6 ^15,16,e^	3.34 ± 0.28 ^35–39,46,q^
RT1500	83.94 ± 6.7 ^9–15,f^	2.26 ± 0.15 ^40–46,r^
T0	121.8 ± 11.6 ^17,19,22^	5.20 ± 0.28 ^47,50,56,k^
T25	126.4 ± 12.1 ^18,20,23^	5.38 ± 0.51 ^51,57,l^
T50	130.5 ± 15.5 ^21,24^	5.47 ± 0.56 ^48,52,58,m^
T100	139.2 ± 18.3	5.54 ± 0.44 ^49,53,59,64,f,n^
T150	143.2 ± 21.7 ^b^	5.30 ± 0.45 ^54,60,h,o^
T250	142.8 ± 20.8 ^25,d^	4.73 ± 0.45 ^55,61,64,i,p^
T500	148.5 ± 13.0 ^17,18,e^	4.42 ± 0.50 ^47–49,62,q^
T1500	149.1 ± 7.1 ^19–21,f^	4.06 ± 0.41 ^50–55,63,r^
T5000	158.9 ± 13.5 ^22–25^	2.26 ± 0.15 ^56–63^

^1–64^*p* < 0.05—statistically significant results within the nanocomposite series. ^a–r^ *p* < 0.05—statistically significant results between nanocomposites, having the same AgNP content, but obtained in different polymerization modes.

## Abbreviations

AgNP	Silver nanoparticles
Bis-GMA	2,2′-bis-[4-(2-hydroxy-3-methacryloyloxy propoxy)phenyl]propane
BPO	Benzoyl peroxide
RT	Room temperature polymerization
CQ	Camphorquinone
*DC*	Degree of conversion
DMPT	*N*,*N*-dimethyl-*p*-toluidine
DMAEMA	*N*,*N*-dimethylaminoethyl methacrylate
E	Flexural modulus
H	Hardness
TEGDMA	Triethylene glycol dimethacrylate
Ph	Photopolymerization
S	Polymerization shrinkage
T	Thermal polymerization
WS	Water sorption
